# Predictive value of ferritin heavy chains in the development of coronary artery calcification in patients on maintenance hemodialysis: a prospective cohort study

**DOI:** 10.3389/fendo.2025.1503940

**Published:** 2025-07-30

**Authors:** Sipei Chen, Yu Tang, Yangmei Pu, Xiaoqiang Xia, Yi Li, Yang Zou

**Affiliations:** ^1^ Department of Nephrology and Institute of Nephrology, Sichuan Provincial People’s Hospital, School of Medicine, University of Electronic Science and Technology of China, Sichuan Clinical Research Centre for Kidney Diseases, Chengdu, Sichuan, China; ^2^ Enyang District People’s Hospital of Bazhong, Bazhong, China; ^3^ School of Medicine, University of Electronic Science and Technology of China, Chengdu, Sichuan, China; ^4^ State Key Laboratory of Oral Diseases, National Clinical Research, Center for Oral Diseases, Chinese Academy of Medical Sciences, Research Unit of Oral Carcinogenesis and Management, West China, Hospital of Stomatology, Sichuan University, Chengdu, Sichuan, China

**Keywords:** vascular calcification, maintenance hemodialysis, ferritin heavy chain, risk factors, predictive value

## Abstract

**Background:**

Vascular calcification (VC) is a well-established risk factor for cardiovascular disease (CVD) and mortality in patients on maintenance hemodialysis (MHD). These patients frequently present with hyperphosphatemia as well as disorders of iron metabolism. This study aims to explore the role of ferritin heavy chain (FTH) in the development and progression of coronary artery calcification (CAC) in patients on MHD and assess its predictive value.

**Methods:**

Using a bioinformatics approach, we analyzed datasets related to VC. In our prospective study, we evaluated the Coronary Artery Calcification Score (CACS) alongside clinical markers, including serum FTH, serum ferritin, and transferrin saturation (TSAT), in patients on MHD at baseline and after a 1-year follow-up.

**Results:**

Fth1 was identified as a differentially expressed gene significantly upregulated in the aorta of both ApoE^-/-^ mice (atherosclerotic calcification model) and chronic kidney disease (CKD) mice (medial calcification model). Among patients on MHD, 85.71% exhibited CAC, with 49.09% showing progression. Patients with CAC tended to be older and have a higher body mass index (BMI). Notably, serum FTH and phosphorus (P) levels were significantly elevated in those with progressive CAC. Elevated serum FTH and high serum P were both independent risk factors for CAC progression and showed predictive value.

**Conclusion:**

Elevated serum FTH and high serum phosphorus are clinically significant predictors of VC progression in patients on MHD.

## 1 Introduction

End-stage renal disease (ESRD) is a major global public health concern. In the United States, data from the National Health and Nutrition Examination Survey (NHANES) indicate that patients with ESRD account for 0.2% of the population, while in China, the number of cases continues to rise (1, 2). Although dialysis can prolong survival in patients with ESRD, their quality of life remains poor, and mortality rates remain high. The annual mortality rate for patients with ESRD is approximately 20%—higher than that of many cancers (3, 4). Among patients with ESRD, those receiving maintenance hemodialysis (MHD) face a particularly high risk, with a 5-year survival rate of just 49%—lower than that of kidney transplant or peritoneal dialysis patients (5, 6). While multiple factors contribute to mortality in ESRD, cardiovascular disease (CVD) is the leading cause, responsible for 23–50% of deaths (5). Thus, reducing CVD incidence is critical to lowering mortality in patients on MHD. Early prediction of CVD is clinically essential to prevent cardiovascular events and improve patient outcomes (7).

Vascular calcification (VC) is recognized as one of the principal causes of CVD in patients with ESRD (8, 9). Data indicate that VC was present in 27% of patients after 1 year of dialysis, with prevalence rising to 83% among those receiving dialysis for over 8 years (10). VC is also regarded as a significant factor increasing both CVD risk and mortality in patients on MHD (11). VC represents a pathobiological process mediated by mechanical damage and influenced by metabolic, endocrine, and inflammatory signaling pathways (12). These processes are associated with vascular smooth muscle cell (VSMC) apoptosis, osteoblast-like differentiation, matrix vesicle release, and extracellular matrix degradation (13). Disruptions in calcium (Ca) and phosphorus (P) metabolism, along with chronic inflammation, oxidative stress, apoptosis, and autophagy, play crucial roles in VC development (14). An imbalance in mineral metabolism can lead to the deposition of Ca and P, which further leads to VC (15). Under pathological conditions, Ca deposition frequently occurs across multiple organ systems, particularly affecting renal, neurological, and cardiovascular tissues (16). Patients with ESRD exhibit specific VC-promoting factors including hyperphosphatemia, uremic toxins, oxidative stress, and chronic inflammation (17). Iron homeostasis disturbances also significantly influence VC progression in patients on MHD. While iron overload induces oxidative stress that drives VC, iron deficiency may contribute through reduced ferritin synthesis and accelerated degradation (18). Notably, VC typically occurs concurrently with abnormal iron homeostasis (19).

Patients on MHD often experience imbalances in iron levels, primarily due to two main factors: absolute iron deficiency and functional iron deficiency. Absolute iron deficiency is typically caused by insufficient intestinal iron intake, impaired absorption, chronic blood loss, and accelerated iron utilization during erythropoiesis-stimulating agent therapy (20). On the other hand, functional iron deficiency is mainly attributed to chronic inflammation and elevated hepcidin levels (21). Ferritin heavy chain (FTH) catalyzes the conversion of Fe²^+^ to Fe³^+^ and stores inert Fe³^+^ in cells, playing a crucial role in regulating iron homeostasis (22). Meanwhile, ferritin light chain (FTL) constitutes the protein shell of ferritin, and the FTH/FTL ratio is dynamically regulated depending on cell type and environment (23). In the context of inflammation and immunity, FTH expression regulation is particularly important for cellular adaptation to iron level fluctuations. FTH expression is influenced by iron at the translational level and by D3T plus inflammatory cytokines at the transcriptional level (24–26). TNF-α can regulate FTH through NF-κB, resulting in its upregulation and serving as an inflammatory biomarker (27). Recent studies suggest that inflammatory cytokines participate in regulating cellular phenotypic transformation, osteogenic differentiation, and VC (28). This implies a potential association between FTH and VC development in patients on MHD, though clinical evidence remains limited. Therefore, this study employed the GEO database for data analysis and applied bioinformatics methods to examine FTH expression. Subsequently, a prospective cohort study was conducted to investigate serum FTH levels in patients on MHD and their correlation with VC progression. The study also aimed to determine whether serum FTH could predict VC occurrence and progression in patients on MHD, potentially providing valuable evidence for early VC intervention and treatment.

## 2 Materials and methods

### 2.1 Bioinformatics analysis

We retrieved gene expression profiling data of mice from the GEO public database (GSE159832, https://www.ncbi.nlm.nih.gov/gds). The data were annotated with gene probes, transformed, and then merged. We corrected batch effects, performed data cleaning, and normalized the data using bioinformatics methods to generate a normalized file. This normalized file was used to identify differentially expressed genes using the R language limma package. The visualization of differential gene intersections from different datasets was performed using VENNY2.1 (https://bioinfogp.cnb.csic.es/tools/venny/). We filtered the gene expression data of Fth1, and analyzed and visualized the protein-protein interaction network of Fth1 using the STRING online database (https://cn.string-db.org/).

### 2.2 Patients

**Figure 1:**
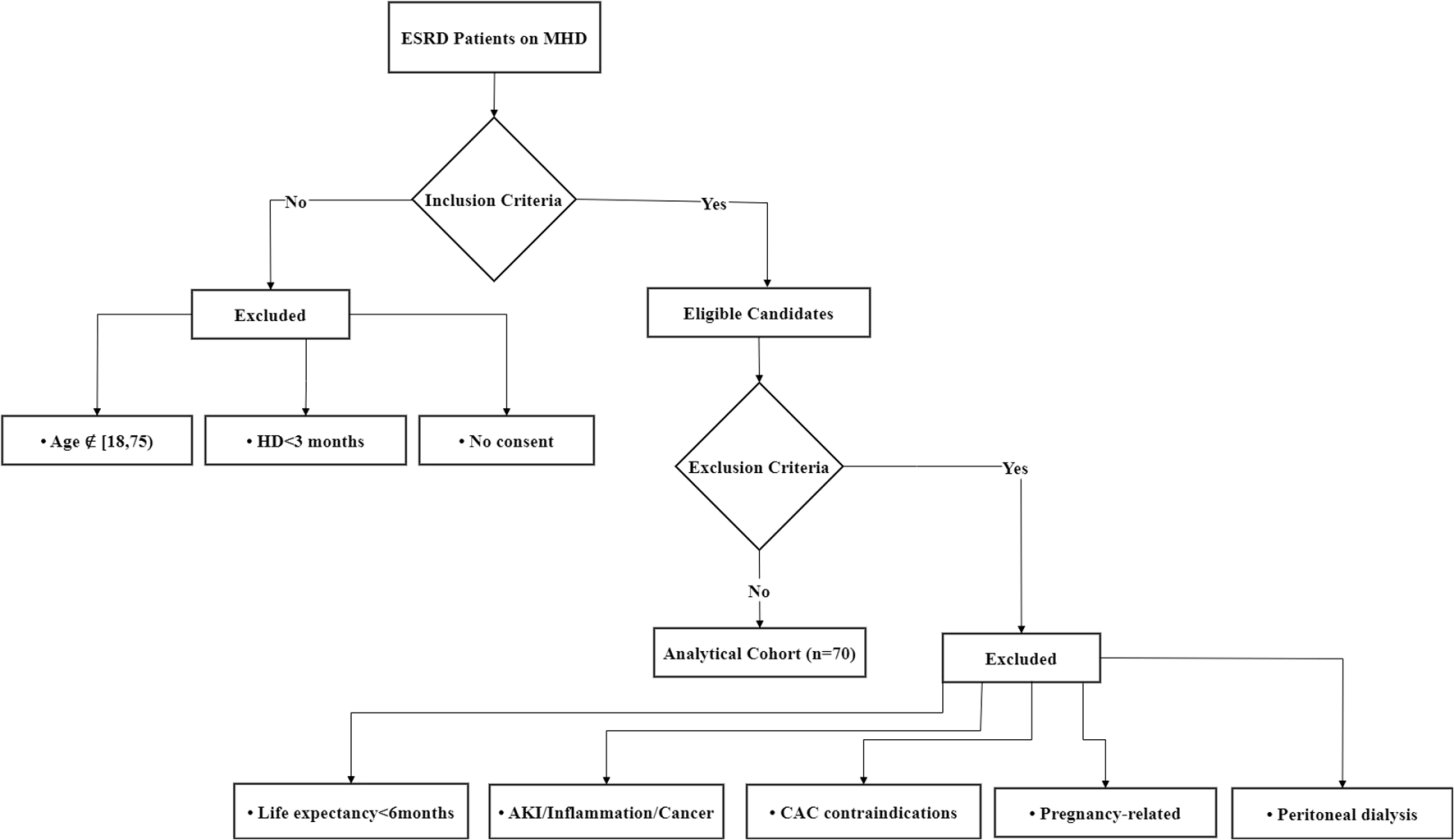
Flow diagram of the patient screening workflow.

This prospective cohort study enrolled patients with ESRD receiving MHD at the Hemodialysis Center of Sichuan Provincial People’s Hospital between January 2022 and December 2022. The inclusion criteria were: (i) age ≥18 and <75 years, regardless of gender; (ii) patients with ESRD receiving stable MHD for ≥3 months; (iii) willingness to provide informed consent by either patients or their legal representatives. The exclusion criteria were: (i) life expectancy <6 months–Ensures that participants can complete the study follow-up and avoids confounding by terminal illnesses that may independently affect cardiovascular outcomes; (ii) presence of acute kidney injury, active inflammatory conditions, or confirmed malignancies–These conditions may independently influence vascular calcification through metabolic disturbances, systemic inflammation, or treatment-related effects (e.g., chemotherapy), potentially confounding the assessment of CAC; (iii) contraindications for CAC testing (e.g., cardiac arrhythmia, stent implantation, amputation, or severe peripheral vascular disease) –These factors may interfere with accurate CAC scoring due to motion artifacts, metal-induced imaging artifacts, or inadequate vascular access for imaging; (iv) current pregnancy, lactation, or planned pregnancy within 6 months–Avoids potential risks of radiation exposure from CAC CT scans and hormonal/pregnancy-related physiological changes that could affect vascular calcification; (v) concurrent peritoneal dialysis treatment–Patients on peritoneal dialysis often have advanced vascular calcification due to chronic kidney disease-mineral and bone disorder (CKD-MBD), which differs mechanistically from calcification in the general population and could bias results. All participants underwent coronary artery multi-slice spiral CT (MSCT) examination. The final cohort comprised 70 patients, and the flow diagram is shown in **Figure 1**.

The study protocol received approval from the Ethics Committee of Sichuan Provincial People’s Hospital (Approval No. 2022-255), with written informed consent obtained from all participants.

### 2.3 Research design

Baseline Visit:

During the baseline visit at study initiation, we collected demographic data, clinical characteristics, and primary disease information, along with performing various tests including complete blood count, blood biochemical parameters, dialysis adequacy assessment parameters, and iron metabolism-related parameters (including ferritin and transferrin saturation [TSAT]). Additionally, 8 mL of fasting whole blood was collected in red-top tubes. The supernatant was separated by centrifugation and stored at -80°C for subsequent ELISA testing to determine serum ferritin heavy chain (FTH; ml024049, Mlbio, China) and hepcidin (E-EL-H6202, Elabscience, China) concentrations. Coronary artery multi-slice computed tomography (MSCT) and coronary artery calcium scoring (CACS) were also completed.

Follow-up Visits:

Each enrolled patient had one scheduled follow-up visit at 12 months (± 7 days). During this visit, complete blood count, blood biochemistry, and iron metabolism tests (ferritin, TSAT) were performed. An 8 mL fasting whole blood sample was collected in a red-top tube, with the supernatant separated by centrifugation and stored at -80°C for serum FTH and hepcidin concentration measurement. Final coronary MSCT and CACS assessments were completed during this visit.

### 2.4 CACS

The CACS was calculated by summing scores from four anatomical sites: the left main artery (LMA), left anterior descending artery (LAD), left circumflex artery (LCX), and right coronary artery (RCA), following the original Agatston method (29). The calcification score for each lesion was determined by multiplying the calcified area by a density factor based on peak CT attenuation values. Each tomographic slice was analyzed individually, with the total score representing the sum of calcification scores from all slices. A CACS of 0 indicated absence of coronary artery calcification (CAC), while any CACS >0 confirmed its presence.

CAC progression was evaluated by comparing 1-year follow-up CACS results with baseline measurements. Progression was defined as either: (i) Transition from no CAC at baseline (CACS=0) to detectable CAC at follow-up (CACS>0), or (ii) Application of the square root (SQRT) method, where a difference of ≥2.5 mm³ between the square root of follow-up CACS and baseline CACS indicated progression.

### 2.5 Statistic analysis

The experimental results were primarily analyzed using statistical methods. Continuous variables with normal distribution were expressed as mean ± standard deviation (SD), while non-normally distributed variables were presented as median (interquartile range, IQR). Categorical variables were expressed as counts (percentages). All statistical analyses were performed using SPSS 22.0 (IBM Corp., Armonk, NY, USA). Two-tailed tests were employed, with statistical significance set at P<0.05, and 95% confidence intervals were used. Baseline characteristics were compared using one-way ANOVA, independent samples t-tests, and chi-square tests, as appropriate. The association between serum ferritin heavy chain (FTH) levels and coronary artery calcification (CAC) progression during the 12-month follow-up was evaluated using ANCOVA for continuous variables and multivariate logistic regression for categorical variables. The predictive value of serum FTH for CAC was determined by calculating the area under the receiver operating characteristic curve (AUC-ROC), along with positive and negative likelihood ratios.

## 3 Results

### 3.1 RNA-seq analysis of calcified aorta

**Figure 2:**
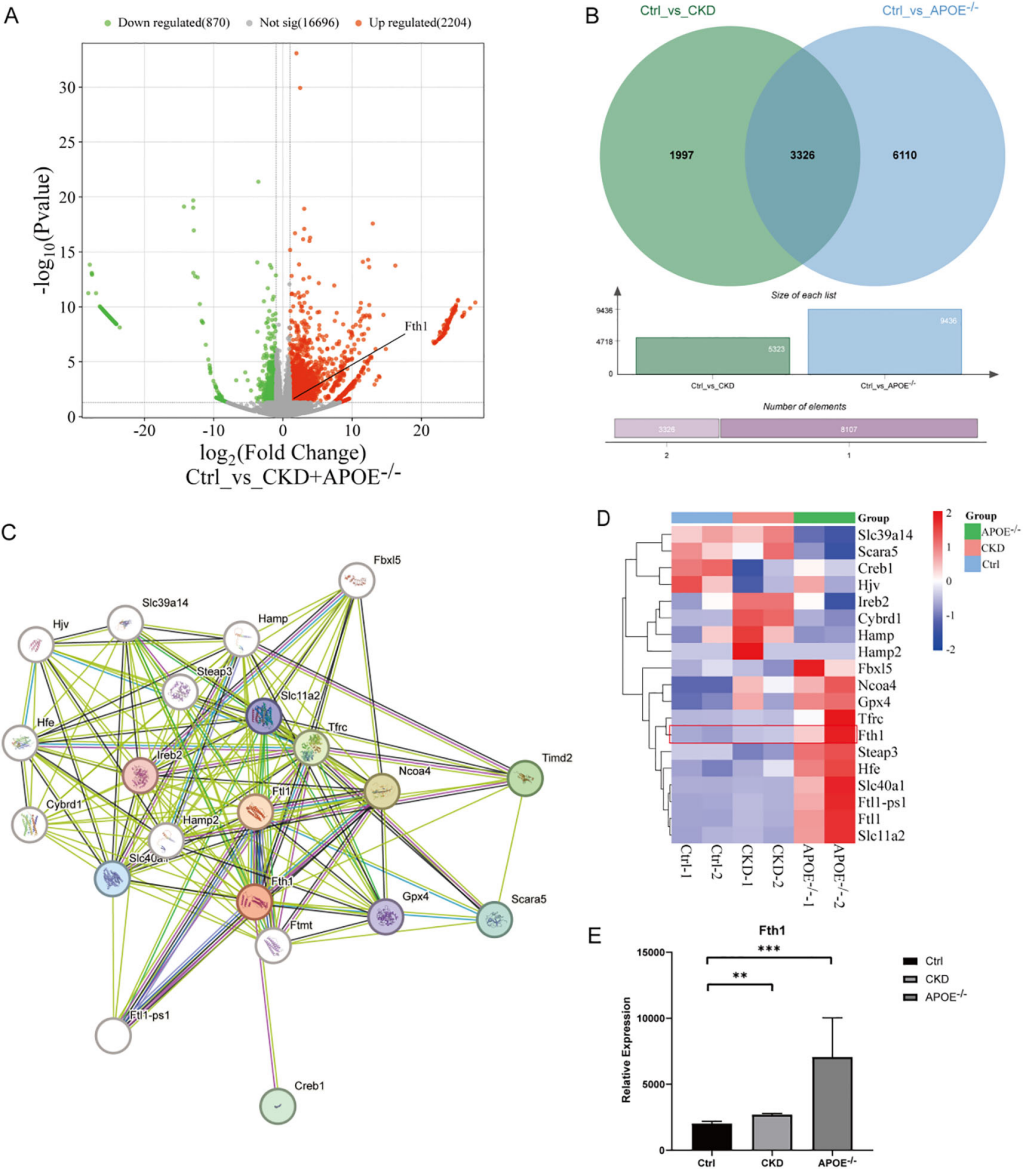
RNA-Seq analysis of aortas from CKD and ApoE^-/-^ calcified mice. DEGs were analyzed using DESeq2 (|log2 fold change| > 1, P < 0.05). **(A)** Volcano plot of DEGs (Ctrl vs. CKD + ApoE^-/-^), with upregulated (red) and downregulated (green) genes highlighted. **(B)** Venn diagram of DEGs overlapping in Ctrl vs. CKD (green) and Ctrl vs. ApoE^-/-^ (blue). **(C)** PPI network of Fth1 and its interactors (Ftl1, Ftl1-ps1, Ncoa4, Tfrc). **(D, E)** Heatmap of Fth1 and associated proteins, along with Fth1 expression levels across groups (histogram). **Statistical significance vs. Ctrl: **P < 0.01, ***P < 0.001. Ctrl, control; CKD, Chronic kidney disease; ApoE^-/-^, Apolipoprotein E knockout mice; DEGs, Differentially Expressed Genes; PPI, Protein-Protein Interaction.

RNA-seq data from aortas of atherosclerotic calcification (ApoE^-/-^ mice) and medial calcification induced by chronic kidney disease (CKD mice) were obtained from the GEO database. DEGs between the control (Ctrl), CKD, and ApoE^-/-^ groups were analyzed using DESeq2. The analysis identified Fth1 as a significantly upregulated gene in both calcified mouse models, as shown in the volcano diagram (**Figure 2A**). Additionally, the Venn diagram (**Figure 2B**) revealed 3326 DEGs shared between the two calcification models. Protein-protein interaction (PPI) network analysis demonstrated that Fth1 interacts with Ftl1, Ftl1-ps1, Ncoa4, and Tfrc, as illustrated in the PPI plot (**Figure 2C**), with interactions supported by established databases and experimental evidence. Furthermore, Fth1 was associated with genes such as Slc11a2, Slc40a1, and Gpx4, as reported in prior studies and predictive analyses. Notably, Fth1 expression was significantly increased in the CKD-induced calcification group and the ApoE^-/-^ atherosclerotic calcification group (**Figures 2D, E**). These findings suggest that Fth1 may play a role in VC development.

### 3.2 Baseline characteristics and CAC in patients on MHD

This study included 70 patients on MHD (55.71% male, 44.29% female) with a mean age of 55.33 ± 11.22 years and mean dialysis duration of 99.63 ± 60.44 months. Primary renal diseases comprised chronic nephritis (33%), diabetic nephropathy (28%), hypertensive nephropathy (26%), and other causes (13%) (**Table 1**).

**Table 1:** Baseline characteristics were compared between patients on MHD with and without CAC.

Variable	Non-CAC group (n=10)	CAC group (n=60)	P value
Sex (male/female)	3/7	36/24	0.096
Age (years)	45.61 ± 6.97	56.94 ± 11.00	0.002*
Dialysis duration (months)	110.00 (55.00, 110.00)	108.00 (53.00, 142.00)	0.425
BMI (Kg/m^2^)	19.51 ± 1.87	22.88 ± 3.72	0.007*
Diabetes [Y/N]	1/9	23/37	0.148
Hypertension [Y/N]	9/1	55/5	1.000
Smoking [Y/N]	3/7	23/37	0.734
Primary disease:	10	60	0.456
Chronic nephritis	5	18	0.216
Diabetic nephropathy	1	19	0.160
Hypertensive nephropathy	3	15	0.738
other	1	8	0.771
Laboratory examination:			
FTH (ng/mL)	60.51 (42.98, 83.47)	46.22 (36.53, 72.48)	0.712
Hepcidin (ng/mL)	29.14 (23.16, 32.52)	28.50 (21.42, 35.02)	0.867
Ferritin (ng/mL)	173.73 (116.69, 648.20)	179.06 (130.22, 232.54)	0.562
Fe (umol/L)	14.92 ± 6.63	14.44 ± 5.29	0.797
TSAT (%)	34.31 ± 14.93	32.91 ± 11.38	0.732
WBC (10^9/L)	5.15 ± 1.47	6.03 ± 1.70	0.127
RBC (10^12/L)	3.46 ± 0.38	3.74 ± 0.61	0.172
Hb (g/L)	105.00 ± 9.90	111.68 ± 12.88	0.123
PLT (10^9/L)	187.90 ± 67.72	172.48 ± 58.56	0.453
Cr (umol/L)	1055.60 ± 193.91	990.38 ± 196.26	0.333
eGFR (ml/min/1.73m^2^)	3.94 ± 0.51	4.25 ± 0.87	0.127
UA (umol/L)	446.70 ± 69.22	427.35 ± 99.14	0.457
BUN (mmol/L)	22.97 ± 4.59	25.09 ± 5.61	0.260
CHOL (mmol/L)	4.37 ± 0.66	3.82 ± 0.95	0.085
TG (mmol/L)	1.61 (1.40, 2.03)	2.02 (1.27, 2.69)	0.476
Glu (mmol/L)	5.76 (4.29, 6.34)	5.79 (4.39, 8.47)	0.290
Na (mmol/L)	138.43 ± 2.12	138.21 ± 3.16	0.834
K (mmol/L)	5.02 ± 0.91	5.01 ± 0.71	0.966
Ca (mmol/L)	2.24 ± 0.21	2.24 ± 0.22	0.944
P (mmol/L)	1.81 ± 0.64	2.01 ± 0.41	0.185
Alb (g/L)	42.21 ± 2.99	42.76 ± 3.07	0.598
ALT (U/L)	9.00 (8.00, 19.00)	10.00 (7.00, 14.00)	0.920
AST (U/L)	10.90 ± 4.31	12.90 ± 5.60	0.286
PTH (pg/mL)	351.00 (204.00, 538.00)	269.00 (171.00, 406.00)	0.953
Baseline medication administration:			
Chalybeate (Y/N)	7/3	31/29	0.281
Statins (Y/N)	4/6	26/34	0.844
Activated vitamin D (Y/N)	8/2	48/12	1.000

CAC prevalence was 85.71% (60/70 patients), with 10 patients (14.29%) showing no calcification (CAC=0). The CACS distribution was non-normal (median: 190.86; mean: 621.12). Calcification involved only 1 vessel (23.33%), only 2 vessels (28.34%), only 3 vessels (30%), or only 4 vessels (18.33%).

Calcification was observed in the LMA in 24 cases (40%; median CACS: 10.11, mean: 53.47), LAD in 45 cases (75%; median CACS: 178.56, mean: 312.27), LCX in 37 cases (61.67%; median CACS: 31.54, mean: 132.08), and RCA in 40 cases (66.67%; median CACS: 124.15, mean: 409.15). The calcification burden varied significantly among coronary arteries, with the LAD and RCA demonstrating both higher prevalence and more severe calcification compared to the LCX and LMA (see **Table 2**).

**Table 2:** CAC distribution in Patients on MHD (n=60).

CAC sites	CACS [M (P25,P75)]	No. of patients involved (%)
LMA	10.11 (3.94,71.64)	24 (40%)
LAD	178.56 (15.22,499.63)	45 (75%)
LCX	31.54 (5.97,263.92)	37 (61.67%)
RCA	124.15 (32.09,511.95)	40 (66.67%)

CAC, coronary artery calcification; CACS, Coronary Artery Calcium Score; M, Median; P25, 25th percentile; P75, 75th percentile; LMA, the left main artery; LAD, left anterior descending artery; LCX, left circumflex artery; RCA, right coronary artery.

All variables are presented as mean ± SD, and median (IQR). BMI: Body mass index; FTH: Serum ferritin heavy chain; Hepcidin: Serum fepcidin; Fe: Serum iron; Ferritin: Serum ferritin; TSAT: Transferrin saturation; WBC: White blood cell; RBC: Red blood cell; Hb: Hemoglobin; PLT: platelet count; Cr: Creatinine; UA: Uric acid; BUN: blood urea nitrogen; CHOL: Cholesterol; TG: Triglyceride; Glu: glucose; Na: Natrium; K: Kalium; Ca: Calcium; P: phosphorus; Alb: Albumin; ALT: glutamic pyruvic transaminase; AST: glutamic oxaloacetic transaminase; ALP: alkaline phosphatase; PTH: parathyroid hormone. *With significant differences.

### 3.3 Comparative analysis of patients on MHD with and without CAC

Patients on MHD were stratified by CACS into CAC (CACS>0, n=60) and non-CAC (CACS=0, n=10) groups for comparative analysis (**Table 1**). The CAC group demonstrated significantly greater age (56.94 ± 11.00, p=0.002) and body mass index (BMI, 22.88 ± 3.72, p=0.007) compared to non-CAC patients. While serum FTH (46.22 (36.53, 72.48), p=0.712) and Hepcidin (28.50 (21.42, 35.02), p=0.867) levels measured by ELISA were numerically elevated in the CAC group, these differences did not reach statistical significance.

### 3.4 Comparison of CAC progression and non-progression in patients on MHD

Among the 70 enrolled patients on MHD, follow-up data were available for 55 patients (due to 4 deaths, 2 renal transplants, and 9 transfers), who underwent repeat MSCT assessments including CACS.

Comparative analysis of 1-year versus baseline CACS revealed CAC progression in 27 patients (49.09% progression rate), with significant differences observed in serum FTH and P levels between groups. Specifically, the CAC progression group exhibited significantly elevated serum FTH (55.82 (44.51, 85.31), p=0.029) levels and P (2.14 ± 0.42, p=0.008) (**Table 3**). Univariate regression analysis identified serum FTH (EXP(B): 1.024, 95%CI: 1.001, 1.046, p=0.037) and P (EXP(B): 5.781, 95%CI: 1.444, 23.151, p=0.013) as risk factors, but not ferritin (**Table 4**). While serum Hepcidin and Ferritin levels were numerically lower in the progression group, the difference did not achieve statistical significance (p > 0.05).

**Table 3:** Comparison between the CAC progression group and the non-CAC progression group.

Variable	Non-CAC progression group (n=28)	CAC progression group (n=27)	P value
Sex (male/female)	14/14	18/9	0.277
Age (years)	55.10 ± 11.13	55.49 ± 10.46	0.895
Dialysis duration (months)	97.50 (55.00, 142.00)	90.00 (44.00, 113.00)	0.409
BMI (Kg/m^2^)	21.71 ± 4.32	23.20 ± 2.88	0.141
Diabetes [Y/N]	8/20	11/16	0.403
Hypertension [Y/N]	27/1	27/0	0.979
Smoking [Y/N]	8/20	12/15	0.269
Primary disease:	28	27	0.156
Chronic nephritis	12	5	0.051
Diabetic nephropathy	6	10	0.203
Hypertensive nephropathy	8	7	0.826
other	2	5	0.206
Laboratory examination:			
FTH (ng/mL)	42.44 (28.02, 60.32)	55.82 (44.51, 85.31)	0.029*
Hepcidin (ng/mL)	30.12 (24.30, 35.88)	29.14 (20.69, 35.02)	0.528
Ferritin (ng/mL)	182.54 (145.53, 347.00)	173.73 (88.42, 200.31)	0.062
Fe (umol/L)	15.06 ± 5.74	13.96 ± 5.06	0.457
TSAT (%)	34.26 ± 12.55	31.27 ± 10.97	0.351
WBC (10^9/L)	5.70 ± 2.08	6.08 ± 0.94	0.384
RBC (10^12/L)	3.67 ± 0.53	3.70 ± 0.53	0.814
Hb (g/L)	109.96 ± 11.10	111.63 ± 13.87	0.624
PLT (10^9/L)	175.07 ± 64.54	170.37 ± 49.13	0.763
Cr (umol/L)	976.57 ± 179.93	1045.36 ± 233.65	0.226
UA (umol/L)	414.39 ± 63.43	449.69 ± 72.23	0.059
BUN (mmol/L)	22.71 ± 3.91	24.89 ± 4.61	0.065
CHOL (mmol/L)	3.98 ± 0.89	3.89 ± 0.98	0.743
TG (mmol/L)	1.97 (1.75, 2.70)	1.90 (1.18, 2.82)	0.485
Glu (mmol/L)	4.92 (4.13, 7.20)	7.05 (4.56, 8.99)	0.115
Na (mmol/L)	138.46 ± 2.76	137.85 ± 3.52	0.477
K (mmol/L)	4.88 ± 0.84	5.14 ± 0.68	0.206
Ca (mmol/L)	2.22 ± 0.22	2.27 ± 0.19	0.317
P (mmol/L)	1.82 ± 0.46	2.14 ± 0.42	0.008*
Alb (g/L)	42.82 ± 2.74	42.63 ± 3.30	0.815
ALT (U/L)	11.00 (7.00, 17.50)	10.00 (8.00, 13.00)	0.408
AST (U/L)	13.00 ± 3.94	12.07 ± 5.64	0.482
PTH (pg/mL)	251.00 (138.75, 351.75)	255.00 (171.00, 459.00)	0.363
Baseline medication administration:			
Chalybeate (Y/N)	14/14	16/11	0.491
Statins (Y/N)	13/15	11/16	0.671
Activated vitamin D (Y/N)	21/7	24/3	0.182

All variables are presented as mean ± SD, and median (IQR). BMI, Body mass index; FTH, Serum ferritin heavy chain; Hepcidin, Serum fepcidin; Fe, Serum iron; Ferritin, Serum ferritin; TSAT, Transferrin saturation; WBC, White blood cell; RBC, Red blood cell; Hb, Hemoglobin; PLT, platelet count; Cr, Creatinine; UA, Uric acid; BUN, blood urea nitrogen; CHOL, Cholesterol; TG, Triglyceride; Glu, glucose; Na, Natrium; K, Kalium; Ca, Calcium; P, phosphorus; Alb, Albumin; ALT, glutamic pyruvic transaminase; AST, glutamic oxaloacetic transaminase; ALP, alkaline phosphatase; PTH, parathyroid hormone. *With significant differences.

**Table 4:** Univariate logistic regression analysis of influencing factors for CAC progression patients on MHD.

Independent variable	EXP (B)	EXP (B) 95%CI	P value
Sex (male/female)	2.375	0.783, 7.203	0.127
Age (years)	1.003	0.955, 1.055	0.892
Dialysis duration (months)	0.996	0.987, 1.005	0.388
BMI (Kg/m^2^)	1.126	0.958, 1.325	0.150
Diabetes [Y/N]	1.719	0.559, 5.285	0.345
Hypertension [Y/N]	0.963	0.057, 16.214	0.979
Smoking [Y/N]	1.450	0.341, 6.178	0.615
Primary disease:			
Chronic nephritis	1.429	0.184, 11.085	0.733
Diabetic nephropathy	4.167	0.607, 28.621	0.147
Hypertensive nephropathy	1.875	0.266, 13.202	0.528
other	6.250	0.615, 63.538	0.121
Laboratory examination:			
FTH (ng/mL)	1.024	1.001, 1.046	0.037*
Hepcidin (ng/mL)	1.000	1.000, 1.000	0.818
Ferritin (ng/mL)	0.996	0.992, 1.000	0.056
Fe (umol/L)	0.962	0.870, 1.064	0.451
TSAT (%)	0.978	0.934, 1.024	0.345
WBC (10^9/L)	0.967	0.877, 1.065	0.490
RBC (10^12/L)	1.133	0.410, 3.132	0.810
Hb (g/L)	1.011	0.968, 1.056	0.618
PLT (10^9/L)	0.999	0.989, 1.008	0.758
Cr (umol/L)	1.002	0.999, 1.004	0.225
UA (umol/L)	1.008	0.999, 1.016	0.066
BUN (mmol/L)	1.130	0.990, 1.289	0.070
CHOL (mmol/L)	0.906	0.509, 1.614	0.738
TG (mmol/L)	0.939	0.601, 1.467	0.782
Glu (mmol/L)	1.059	0.929, 1.206	0.391
Na (mmol/L)	0.938	0.790, 1.115	0.470
K (mmol/L)	1.588	0.777, 3.246	0.205
Ca (mmol/L)	4.075	0.265, 62.603	0.313
P (mmol/L)	5.781	1.444, 23.151	0.013*
Alb (g/L)	0.979	0.819, 1.169	0.811
ALT (U/L)	0.938	0.850, 1.035	0.205
AST (U/L)	0.960	0.857, 1.075	0.477
PTH (pg/mL)	1.000	0.998, 1.002	0.875
Baseline medication administration:			
Chalybeate (Y/N)	1.455	0.501, 4.227	0.491
Statins (Y/N)	0.793	0.273, 2.308	0.671
Activated vitamin D (Y/N)	2.667	0.611, 11.643	0.192

BMI, Body mass index; FTH, Serum Ferritin heavy chain; Fe, Serum iron; Ferritin, Serum ferritin; TSAT, Transferrin saturation; WBC, White blood cell; RBC, Red blood cell; Hb, Hemoglobin; PLT, platelet count; Cr, Creatinine; UA, Uric acid; BUN, blood urea nitrogen; CHOL, Cholesterol; TG, Triglyceride; GLU, glucose; Na, Natrium; K, Kalium; Ca, Calcium; P, phosphorus; Alb, Albumin; ALT, glutamic pyruvic transaminase; AST, glutamic oxaloacetic transaminase; ALP, alkaline phosphatase; PTH, parathyroid hormone. *With significant differences.

### 3.5 Independent risk factors for CAC progression

Multivariable logistic regression analysis incorporating serum FTH and P (both significant in univariate analyses), along with Ca (a previously established contributor to CAC progression) and established cardiovascular risk factors (sex, BMI, and diabetes), identified serum FTH (EXP(B): 1.025, 95%CI: 1.001, 1.050, p=0.045) and P (EXP(B): 5.045, 95%CI: 1.025, 24.837, p=0.047) as independent predictors of CAC progression (**Table 5**).

**Table 5:** Multivariate logistic regression analysis of CAC progression risk factors in patients on MHD.

Independent variable	B	EXP (B)	EXP (B) 95%CI	P value
Sex (male/female)	0.913	2.492	0.657, 9.453	0.179
BMI (Kg/m^2^)	0.007	1.007	0.845, 1.201	0.935
Diabetes [Y/N]	0.177	1.193	0.303, 4.701	0.800
FTH (ng/ml)	0.025	1.025	1.001, 1.050	0.045*
P	1.618	5.045	1.025, 24.837	0.047*
Ca	1.671	5.316	0.263, 107.634	0.276

BMI, Body mass index; FTH, Serum Ferritin heavy chain; P, phosphorus; Ferritin, Serum ferritin; Ca, Calcium. *With significant differences.

### 3.6 Prediction of CAC progression by serum FTH, ferritin, and P

**Figure 3:**
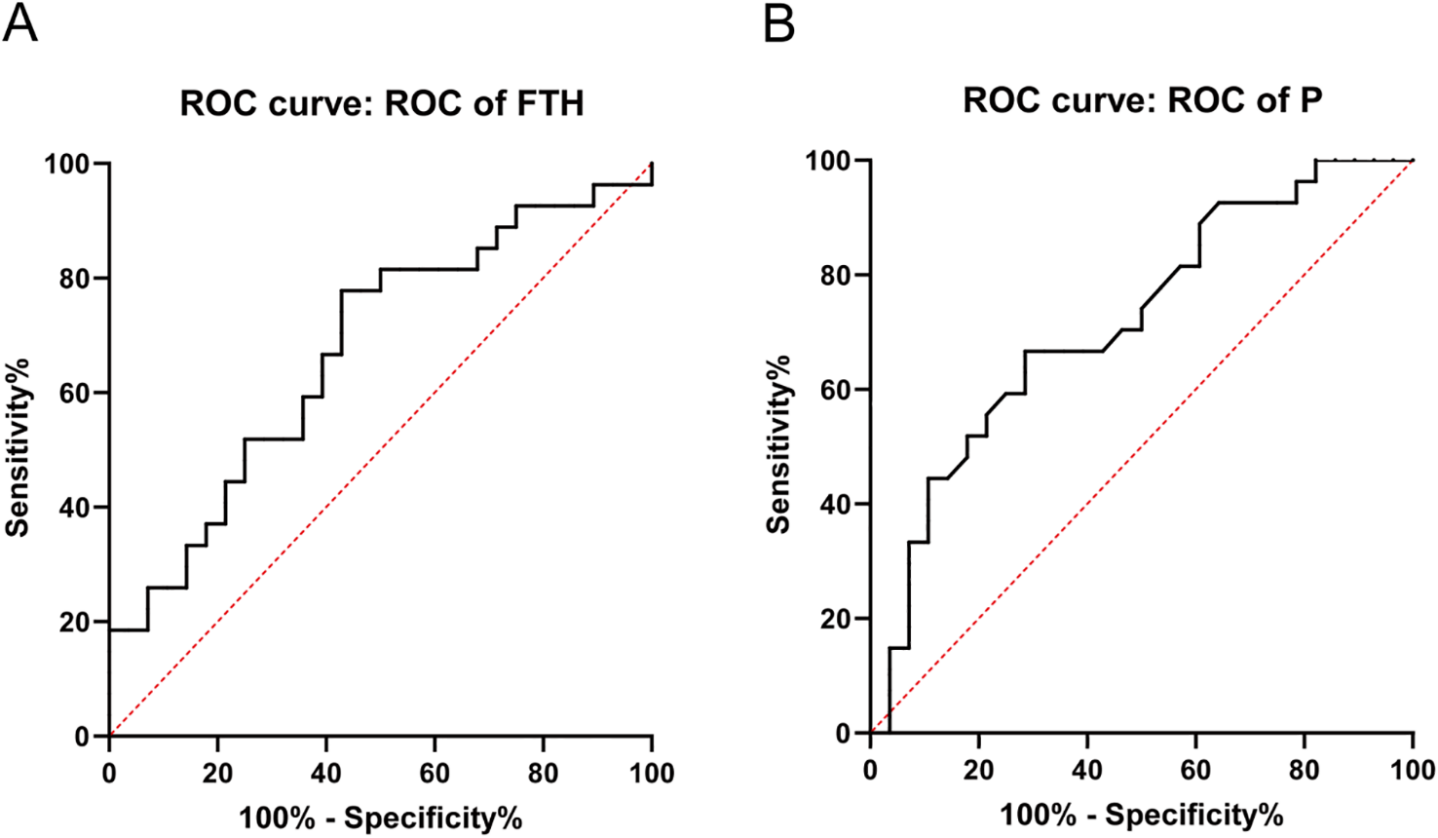
Prediction of CAC progression by serum FTH and P. **(A)** ROC curve for serum FTH. **(B)** ROC curve for serum P. FTH, Ferritin heavy chain; P, phosphorus.

ROC curve analysis evaluated the predictive accuracy of serum FTH and P for CAC progression (**Figure 3**). The area under the curve for serum FTH was 0.672 (P=0.029), with an optimal threshold of 44.46 ng/mL, demonstrating a sensitivity of 77.78% and a specificity of 57.14% (95% CI: 0.529-0.815). Serum P showed an area under the curve of 0.717 (P=0.006), with an optimal threshold of 1.960 mmol/L, yielding a sensitivity of 66.67% and a specificity of 71.43% (95% CI: 0.581-0.853).

## 4 Discussion

CKD represents a major global public health concern, affecting approximately 9-13% of the global population (30, 31), including 8.2% of China’s population based on a cohort of 176,874 subjects (32). As the 7th leading cause of global mortality (33) CKD is particularly concerning due to its association with VC - a well-established risk factor for CVD and related mortality, especially among patients with ESRD receiving MHD (34). Indeed, VC serves as a strong predictor of CVD and all-cause mortality in patients on MHD, driven by dialysis vintage, abnormal mineral metabolism, uremic toxin accumulation, inappropriate use of calcium-containing medications or vitamin D analogs, disrupted calcification inhibitors, oxidative stress, inflammation, anemia, and apoptosis (35). Supporting this, the China Dialysis Calcification Study (CDCS) reported a 72.4% prevalence of CAC - the most common VC manifestation in patients on MHD, who experience more severe, rapidly progressive VC with worse clinical outcomes (36). In our study of 70 patients on MHD, we observed an 85.71% CAC prevalence (60/70 patients; CACS>0), with calcification severity varying significantly across coronary arteries: most severe in the LAD and RCA, and least severe in the LMA. The CAC group was significantly older and had higher BMI, though dialysis vintage, diabetes/hypertension prevalence, and smoking history did not differ significantly. Notably, after 1-year follow-up (with 55 patients remaining after 4 deaths, 2 transplants, and 9 transfers), we identified a 49.09% CAC progression rate, independent of demographic/clinical characteristics or medication use. These findings objectively demonstrate the high prevalence and progression rate of CAC in patients on MHD.

Emerging evidence suggests VC resembles an actively regulated osteogenic process, mediated through multiple mechanisms including dysregulated Ca and P metabolism, impaired calcification inhibitors, secondary hyperparathyroidism, and genetic/hormonal factors (37). This complex process involves intricate interactions among VSMCs, endothelial cells (ECs), mesenchymal stem cells (MSCs), calcifying vascular cells (CVCs), and macrophages (38). Key clinical risk factors include hypertension, diabetes mellitus, dyslipidemia, and hyperuricemia, while pathological drivers encompass inflammation, hyperphosphatemia, uremic toxins, and deficiency of calcification inhibitors (e.g., fetuin-A, matrix Gla protein [MGP], and pyrophosphate) that promote VSMC transdifferentiation into osteoblast-like cells (39). Particularly relevant to patients with ESRD, hyperphosphatemia represents both a hallmark of metabolic disturbance and an independent predictor of CAC (17). Progressive renal dysfunction in CKD leads to Ca/P homeostasis disruption, uremic toxin accumulation, and chronic vascular inflammation, collectively driving VSMC phenotypic transformation (40). Elevated phosphate levels play a pivotal role in VC pathogenesis: by inducing VSMC-derived matrix vesicle release, which serve as nucleation sites for Ca/P deposition, thereby promoting irreversible osteogenic differentiation (41). Clinically, hyperphosphatemia correlates with increased cardiovascular mortality and peripheral artery disease risk in patients on MHD (42). Our study demonstrated that while baseline serum P levels did not differ significantly between non-CAC and CAC groups (p>0.05), they were elevated in CAC patients. Longitudinal analysis revealed significantly higher phosphate levels in CAC progressors (p<0.05), with multivariable analysis identifying hyperphosphatemia (>1.960 mmol/L) as an independent predictor of CAC progression. These findings underscore the critical importance of rigorous phosphate monitoring and control in patients on MHD to mitigate VC development and progression.

Growing evidence implicates dysregulated iron metabolism as a key contributor to VC in CKD patients (18). Patients on MHD frequently develop iron deficiency due to impaired intestinal absorption, chronic blood loss (from both gastrointestinal sources and dialysis procedures), and increased iron demands from erythropoiesis-stimulating agent (ESA) therapy (43). These patients often develop renal anemia - defined as hemoglobin <13 g/dL (men) or <12 g/dL (women) - due to insufficient erythropoietin production (44). While intravenous/oral iron supplementation is required to maintain adequate iron stores, excessive administration combined with frequent blood transfusions may cause iron overload (45). Current clinical practice relies on serum ferritin and TSAT to monitor iron status (46). Ferritin is the primary protein for storing iron in the human body and is tightly regulated by iron levels (47). Ferritin has a highly conserved three-dimensional structure, a spherical hollow shell of 24 subunits that can store up to 4500 Fe^3+^. These subunits consist of FTH and FTL with molecular weights of 19 and 21 kDa, respectively. They are synthesized in specific stable ratios in particular cell types during differentiation (22).

FTH exhibits ferroxidase activity, catalyzing the conversion of Fe^2+^ to Fe^3+^ and enabling the storage of inert Fe^3+^ in the iron core (48). FTL does not have Fe-oxidase activity and primarily facilitates Fe nucleation, mineralization, and long-term Fe storage (49). High iron concentrations were specific for FTL, leading to a significant increase in its mRNA levels, while FTH gene transcription remained unaffected (50). FTH gene transcription is primarily regulated by inflammation, oxidative stress, and cytokines. Both proinflammatory cytokines and tumor necrosis factor increase FTH mRNA levels but do not alter FTL gene transcription (51). Thus, FTH acts as a preferential up-regulator of acute phase reactants over FTL during inflammatory conditions (52). In addition, FTH is implicated in the initiation and advancement of VC. Aierken et al. discovered that calcified blood vessels exhibited increased expression of both FTH and the osteogenic protein BMP2. VSMCs treated with Ca and P also displayed elevated expression levels of FTH, Runx2, and BMP2 (53). Yuan et al.’s study of ApoE^-/-^ atherosclerotic mice observed higher deposition of Fth1, Ftl, and TfR1 in the abdominal aorta and increased expression of their Nrf-2 (54). Similarly, *klotho* mutant (*kl/kl*) mice with arterial calcification showed increased Fth1 expression in arterial smooth muscle cells (SMCs) (55). In the present study, bioinformatics analysis of the GSE159832 dataset uncovered that Fth1 is a differentially expressed gene which is significantly upregulated in both ApoE^-/-^ and CKD aortic calcification mice. Fth1 may also have interactions with genes such as Ftl1, Ftl1-ps1, Ncoa4, Tfrc, Slc11a2, Slc40a1, and Gpx4. Nuclear receptor coactivator 4 (NCOA4), a major regulator of ferritin autophagy, directly binds FTH and transfers the complex to the autolysosome for degradation and release of stored iron (56). The transferrin receptor (TFRC) is key in regulating iron uptake (57). Solute carrier family 11, member 2 (SLC11A2) is likewise important for cellular iron uptake (58). Glutathione peroxidase 4 (GPX4) protects cells from oxidative damage by reducing oxidative substrates (59). The majority of current studies on the relationship between FTH and VC involve animal or cellular models, with very few focusing on patients on MHD. In our one-year prospective study, we discovered that serum FTH levels were significantly higher in patients with progressing CAC (P<0.05), indicating it as an independent risk factor for this progression. It is hypothesized that the role of FTH in the progression of CAC is closely linked to VC due to inflammation and oxidative stress, as these factors tend to increase FTH levels rather than FTL. However, further research is necessary to confirm this hypothesis.

Our study has some limitations. First, the study population was limited to specific healthcare organizations and a specific time period, which may introduce bias and impact the generalizability of the findings. Second, the sample size was small, raising the possibility of overfitting in multifactor regression analysis. Lastly, the limited sample size may have affected the validity of statistics and the results of the area under the ROC curve. However, indicators such as serum FTH still demonstrate good predictive value (P<0.05). We anticipate that larger clinical studies in the future will help confirm these findings.

In summary, elevated serum P levels in patients on MHD can lead to VSMC damage, which may contribute to the progression of CAC. Additionally, elevated serum FTH levels may be influenced by inflammation or infection in patients on MHD and also play a role in the progression of CAC. Therefore, this study aimed to identify the risk factors for the progression of CAC in patients on MHD and to identify serological markers with good efficacy that can help predict and diagnose the progression of VC at an early stage. These markers can guide clinicians in choosing appropriate medications to treat or slow down the progression of VC, ultimately reducing the incidence of CVD and the mortality rate in patients on MHD.

## Data Availability

The original contributions presented in the study are included in the artical/supplementary material, further inquiries can be directed to the corresponding authors.
